# Use of quantile regression to investigate changes in the body mass index distribution of Chinese adults aged 18–60 years: a longitudinal study

**DOI:** 10.1186/s12889-015-1606-8

**Published:** 2015-03-21

**Authors:** Yifei Ouyang, Huijun Wang, Chang Su, Zhihong Wang, Yiqi Song, Yingting Xiao, Wenwen Du, Bing Zhang

**Affiliations:** National Institute for Nutrition and Health, Chinese Center for Disease Control and Prevention, Beijing, China; Department of Physical Education, North China Institute of Science and Technology, Hebei, China

**Keywords:** China, Body mass index, Quantile regression, Trend

## Abstract

**Background:**

Traditional linear regression analyses have detected increasing trends in the incidence of overweight/obesity among both genders in China. However, these previous regression analyses were limited in their ability to capture cross-distribution variations among effects. The objective of our study was to analyze the change in the body mass index (BMI) distribution of adults and investigated the relationships between the key covariates and the BMI distribution.

**Methods:**

We used longitudinal data from the China Health and Nutrition Surveys (CHNS) in 1991, 1993, 1997, 2000, 2004, 2006, 2009, and 2011, with at least two waves of data collection. In total, 17,819 participants aged 18–60 years (N = 8587 men and 9232 women) were included in the final analysis with 48,900 observations. The lambda-mu-sigma (LMS) method was used to describe changes in the BMI distribution. Separate sex-stratified longitudinal quantile regression (QR) analyses were used to investigate changes in the BMI distribution over time.

**Results:**

The main characteristics of the BMI changes in both genders were that the curves shifted to the right and the distributions became wider. All of the BMI percentile curves tended to increase from 1991 to 2011, where the levels increased more in the higher percentiles. The QR analyses showed that these patterns remain consistent after adjusting for individual and community level factors. Physical activity (PA) had a negative association with BMI for both genders in all percentiles. Income and energy intake were associated with positive changes in male BMI in the upper percentile. Sedentary time had a positive association with female BMI in the middle percentile. Compared with less educated women, women with senior school education at 75^th^ percentile had 0.951 kg/m^2^ lower BMIs.

**Conclusions:**

This longitudinal quantile regression suggests that effects of different covariates worked differently across the BMI distribution. Since social and economic characteristics in China have underlined the significant disparities in many aspects, national strategies to tackle overweight/obesity should be tailored as appropriate for various segments.

## Background

Traditionally, obesity is associated with developed countries but it has now become a pandemic throughout the world, where this health burden has emerged in developing countries, such as China [1-4]. According to the Chinese Chronic Disease Surveillance study in 2010, the prevalence of overweight and obese Chinese adults increased rapidly in the last decade, reaching 30.6% and 12.0% in 2010, respectively [5]. No countries have obtained significant decreases in the rate of obesity in the last three decades [6], and being overweight/obese is one of five leading global risks for mortality throughout the world [7]. The economic costs of overweight and obese adults have increased recently in China. In 2010, overweight/obese adults imposed a substantial economic burden on China, where they were responsible for 42.9% of the medical and non-medical yearly costs of the major non-communicable and chronic diseases (NCDs) in China [8].

The obesity epidemic is attributed to a widespread imbalance between energy intake and energy expenditure [9]. China has experienced dramatic economic and social changes in the past decades, and the pace of urbanization in China is much faster than that experienced previously in Western countries. These changes have affected the lifestyles of Chinese people, especially the way they eat, drink, and move. Thus, the Chinese population have rapidly increased their consumption of edible oils, animal-derived foods, snacks, and fried foods, and many have adopted other unhealthy behaviors [10]. At present, eating away from home is playing a larger part in the Chinese diet due to rapid urbanization and income growth. Furthermore, the modern food system is producing more processed, affordable, and easily accessible foods and beverages than ever before [11]. As a result of all these factors, China’s history of under-nutrition has been followed by a rapid increase in obesity and obesity-related diseases, with differential rates across rural and urban areas [12,13]. Global urbanization has provided many benefits in terms of living, transportation, health services, and other facilitative community aspects, but rapid urbanization has produced deleterious effects on the eating habits, lifestyles, and overall health of individuals within developing communities, thereby causing a significant nutritional transition, particularly in Africa, China, and India [14]. A recent study demonstrated that rapid changes in mechanized transportation, home production, and market production have contributed to a dramatic reduction in total PA in China [15]. A cross sectional analysis from Bhavani showed energy intake had largely insignificant effect on BMI in the lower and middle quantiles, whereas the upper quantiles show a positive and significant effect [16]. Several studies have investigated a number of individual-level effects on overweight Chinese adults, such as the educational attainment disparities [17] and regional differences [3]. Researchers have also attributed a major role to urbanization in the obesity epidemic [11,18,19].

However, previous researchers assessed the frequency of obesity trends by focusing on the upper percentiles of the BMI frequency distribution in categorical logistic regression analyses or other traditional linear regressions [20,21]. These approaches are limited because the effect of obesity-related covariates can be hypothesized to vary across the BMI distribution, thereby preventing us from fully understanding the causes of the shift [22]. The application of QR analysis rather than traditional regression is particularly helpful when the effect of covariates differ at different levels of the response variable. Therefore, we analyzed the shifts in adult BMI distributions and modeled the influence of predictors on changes in the 10^th^, 25^th^, 50^th^, 75^th^, and 90^th^ BMI percentile over time using data obtained from the CHNS. The results of this study provide important insights that may facilitate the control, prevention, and treatment of overweight/obesity by health decision makers.

## Methods

### Study design

The data were derived from the CHNS, which is a prospective household-based study that included individuals of various ages in nine rounds of surveys between 1989 and 2011, who lived in nine diverse provinces and three mega-cities (only in 2011). The CHNS focused on assessing the relationships between the economic, sociological, and demographic transformation of China, and the resulting effects on the health and nutritional status of the Chinese population. A multistage, stratified, sampling design was used to ensure that the CHNS provided a fair representation of urban and rural areas. The data were collected by trained and certified health workers. The survey protocols, instruments, and the process used to obtain informed consent in this study were approved by the institutional review committees of the University of North Carolina at Chapel Hill, as well as by the National Institute for Nutrition and Health, which is affiliated with the China Center for Disease Control and Prevention. The participants provided written, informed consent. Additional details regarding the CHNS data have been published previously [23].

### Study population

Our samples included the eight most recent rounds of the CHNS, which were collected in 1991, 1993, 1997, 2000, 2004, 2006, 2009, and 2011, with at least two waves of data collection to facilitate the assessment of temporal trends in these individuals. Data from 1989 were not included because some of the covariates, such as PA and sedentary time, were not obtained during these waves. We limited the sample to subjects aged between 18 and 60 years who were not disabled, pregnant, or lactating during a particular wave, and complete data were available for these participants. We also excluded all of the observations from the three megacities because these data were only collected in 2011. Our final analysis sample included 17,819 participants (N = 8587 men and 9232 women) and 48,900 observations, i.e., an average of approximately 2.7 observations were collected for each subject.

### Outcomes

The outcome of interest was BMI (kg/m^2^), which was calculated as the weight (in kg) divided by the height (in m) squared. Weight and height were measured by trained field staff according to the standardized protocols of the World Health Organization. Weight was measured without shoes and in light clothing to the nearest 0.1 kg using a calibrated beam scale. Height was measured without shoes to the nearest 0.1 cm using a portable stadiometer. According to the Chinese definitions, overweight and obesity were defined based on BMI cutoff points of 24 and 28, respectively. We used BMI as a continuous variable.

### Covariates

Two types of covariates were used in the analysis. The first type comprised categorical variables. An individual’s education was determined as the highest educational level reported throughout the course of inclusion in the CHNS. Educational level was categorized into three groups: 0 = middle school education or less; 1 = high school education; 2 = college education and above. Individual income was based on the reported gross annual per capita household income, which was inflated to 2011 values [24] and categorized into wave-specific tertiles.

The second type comprised continuous variables. Detailed dietary consumption data were collected at both the household and individual level over three consecutive days to determine the average daily energy intake for each individual [25,26]. According to previous studies of the CHNS [16,27], the vast majority of the PA among adults in China comprises occupational and domestic PA, whereas the amount of leisure and travel PA is extremely low. Moreover, the occupational and domestic PA components were the only types for which we had complete data across all eight waves. Thus, we used the average metabolic equivalents of task (MET) hours per day to indicate the PA level, which comprised two PA domains: occupational and domestic. A MET unit is defined as the ratio of a person’s working metabolic rate relative to their resting (basal) metabolic rate. Therefore, the average MET-hours per day measurements comprise both the average intensity of each activity (or sub-activity) and the time spent in each activity. Measurements of the sedentary leisure time among adults were only available from 2004 in the CHNS study. Sedentary behavior was calculated as the average hours per day because low MET values are associated with sedentary activity. The calculation of these values has been described in previous studies [27,28].

A multidimensional index was developed specifically for the CHNS to capture the urbanization level in Chinese communities [3]. This index is a measure of various dimensions of urbanization and it has been used in previous studies [27,29,30]. In the present study, we divided the urbanization index into tertiles: 0 = low urbanization, 1 = medium urbanization, and 2 = high urbanization. Age was defined as the age in 2011.

### Statistical analysis

First, we calculated descriptive statistics for the individual demographic variables, which were stratified by gender in each wave of the survey. Continuous variables were expressed as medians, the 25^th^ percentile (Q1), and the 75^th^ percentile (Q3). Categorical variables were expressed as percentages. To examine the different changes over time, we used trend Chi-square tests for categorical variables and Kruskal–Wallis tests for continuous variables. *P* < 0.01 were considered statistically significant.

**Figure 1:**
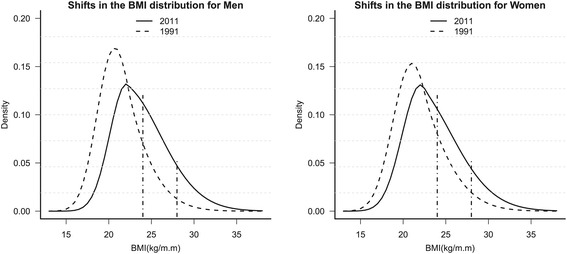
**Shifts in the BMI distribution for Chinese adults aged 18–60 years old in 1991–2011.**

**Figure 2:**
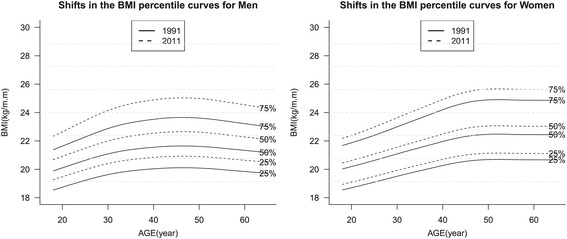
**Shifts in the BMI percentile curves for Chinese adults aged 18–60 years old in 1991–2011.**

Second, we used the LMS method in the VGAM package R version 2.15.1 (R Development Core Team, Vienna, Austria) to obtain figures to represent the BMI distributions in 1991 and 2011, thereby illustrating the changes in the obesity measures (Figure 1). In addition, we used the LMS method to determine the age-specific secular trends in the obesity measures, thereby allowing us to examine the temporal trends in specific percentile points of the BMI (Figure 2).

Finally, separate sex-stratified longitudinal QR analyses were used to investigate the changes in the BMI distribution over time. Compared with the traditional linear regression based on means, this method is helpful when the effects of covariates vary at different levels of the response variable. This technique also allowed us to consider repeated measurements collected for the same individual over time, to handle the skewed distribution of the outcome variable, and to describe the overall distribution [9]. We aimed to determine whether the changes in the BMI distribution over time were due to secular trends, or other individual-level and community-level factors.

We considered three models, as follows. Model 1 was controlled for time only, thus the coefficients indicated the change in the BMI associated with each additional year for a particular percentile of the BMI distribution. The individual-level variables were then added to the equation to estimate Model 2, including the educational level, per capita annual family income, energy intake, PA, and sedentary hours. For Model 3, the community-level variable was also introduced into Model 2. The descriptive and QR analyses were conducted in lqmm package R version 2.15.1 (R Development Core Team, Vienna, Austria).

## Results

The characteristics of the study samples are shown in Table 1.

**Table 1:** **Demographic characteristics of samples**

**Wave**	**1991**	**1993**	**1997**	**2000**	**2004**	**2006**	**2009**	**2011**	***P*** ^***1***^
Sample size (N)	5870	6165	5955	6777	6439	6286	6121	5287	
Age	36.83 (28.36,44.69)	38.09 (29.56,46.22)	39.99 (31.21,47.69)	41.54 (33.14,49.03)	43.27 (35.24,51.30)	44.05 (36.70,52.13)	45.61 (38.08,53.11)	47.13 (39.89,54.09)	<0.01
Education level (%)									<0.01
None/primary/junior middle school	97.94	98.27	97.95	96.05	95.99	94.45	94.47	91.88	
Senior school	2.00	1.68	2.03	3.89	4.00	5.53	5.48	7.88	
College and above	0.07	0.05	0.02	0.06	0.02	0.02	0.05	0.25	
Income tertile									0.85
Low income	34.49	33.40	33.82	33.18	33.33	33.20	32.99	33.63	
Middle income	32.93	33.24	32.99	33.78	33.69	33.63	33.36	33.05	
High income	32.58	33.36	33.19	33.03	32.98	33.17	33.66	33.32	
Energy intake (1000 kcal/day)	24.47 (19.94,29.05)	23.75 (19.63,28.36)	24.54 (20.44,29.63)	23.56 (19.28,28.17)	22.76 (18.61,27.68)	22.98 (18.64,28.40)	21.80 (17.88,26.39)	20.77 (16.48,25.79)	<0.01
Total physical activity (METs/d)	60.86 (31.48,90.68)	51.77 (28.57,80.57)	50.66 (27.40,77.15)	43.57 (19.89,68.57)	32.95 (14.77,59.00)	33.20 (15.14,57.35)	32.29 (15.59,56.96)	31.05 (15.79,55.71)	<0.01
Sedentary time (hours/d)	-	-	-	-	2.00 (1.33,3.50)	2.00 (1.50,3.30)	2.29 (1.64,3.57)	2.14 (1.65,3.29)	<0.01
Urbanization index	43.92 (31.91,60.57)	45.21 (33.37,62.54)	51.12 (35.18,66.90)	55.19 (42.93,76.76)	58.75 (44.51,81.63)	62.24 (47.41,82.48)	64.09 (50.86,84.69)	68.05 (52.25,86.54)	<0.01
BMI (kg/m^2^)	21.28 (19.74,23.16)	21.52 (19.95,23.47)	21.91 (20.20,24.09)	22.54 (20.63,24.84)	22.81 (20.76,25.16)	22.90 (20.99,25.29)	23.22 (21.13,25.61)	23.55 (21.40,25.97)	<0.01

The values are expressed as medians (Q1, Q3) or percentages (%).

*P* ^*1*^ < 0.01, trend Chi-square tests for categorical variables and Kruskal–Wallis tests for continuous variables.

### Trends in the BMI distribution curves of men and women

Figure 1 shows the smoothed distribution curves obtained using the LMS method for the changes in the BMI over time among men and women aged 18–60 years in selected years. For both genders, the main characteristic was a shift to the right by both curves where the distributions became wider and a large proportion of the samples had a higher BMI. During the overall period, the distribution curve moved greatly for men but only slightly for women.

### Shifts in the 25^th^, 50^th^, and 75^th^ percentile BMI curves

Figure 2 shows the 25^th^, 50^th^, and 75^th^ percentile BMI curves constructed using the LMS method for selected years, by gender and age. The 25^th^, 50^th^, and 75^th^ percentile curves are shown from the bottom to the top (solid line: 1991; dotted line: 2011), respectively. All of the percentile curves exhibited increasing trends from 1991 to 2011, where the levels increased more in the higher percentile levels among both genders and each age group. A comparison of the plots shows that the BMI percentile curves of women were more stable than those of men. For each percentile curve, the changes were lower in women than in men. However, after 50 years of age, all of the male BMI percentiles began to decline whereas the female BMI percentiles continued to increase.

### Yearly change in BMI percentiles based on the longitudinal QR analysis

Table 2 shows the yearly change in the BMI percentiles among men and women based on the longitudinal QR analysis. The results obtained with Model 1 suggest that there were significant increases from the 10^th^ percentile to the 90^th^ percentile. These increases were greater in the upper percentiles compared with the lower percentiles. For example, in the 90^th^ percentile, the BMI increased by 0.126 kg/m^2^ (95% CI: 0.117, 0.134) and 0.127 kg/m^2^ (95% CI: 0.118, 0.136) in men and women, respectively. These patterns remained consistent even after adjusting for individual level factors, as shown by the results obtained with Model 2. However, after adjusting for urbanicity (Model 3), the increase only attenuated noticeably for men.

**Table 2:** **Quantile regression results for the different percentiles based on the yearly coefficients (95% CI** ^**1**^ **)**

**Year effects on**			**Coefficient**		
	**10th**	**25th**	**50th**	**75th**	**90th**
Male					
Intercept	18.891 (17.669,20.113)^2^	18.744 (17.917,19.571)^2^	19.680 (18.988,20.372)^2^	20.617 (19.941,21.294)^2^	22.93 (22.016,23.836)^2^
Model1	0.101 (0.089,0.113)^2^	0.109 (0.102,0.117)^2^	0.118 (0.111,0.125)^2^	0.126 (0.118,0.133)^2^	0.126 (0.117,0.134)^2^
Model2	0.074 (0.050,0.098)^2^	0.098 (0.079,0.118)^2^	0.113 (0.092,0.133)^2^	0.104 (0.085,0.122)^2^	0.118 (0.087,0.150)^2^
Model3	0.070 (0.036,0.103)^2^	0.096 (0.074,0.118)^2^	0.102 (0.082,0.121)^2^	0.094 (0.073,0.115)^2^	0.098 (0.064,0.133)^2^
Female					
Intercept	16.075 (0.077,0.107)^2^	17.300 (0.103,0.121)^2^	18.827 (17.584,20.071)^2^	19.205 (18.338,20.072)^2^	19.746 (18.707,20.785)^2^
Model1	0.092 (0.077,0.107)^2^	0.112 (0.103,0.121)^2^	0.115 (0.108,0.121)^2^	0.121 (0.113,0.129)^2^	0.127 (0.118,0.136)^2^
Model2	0.072 (0.051,0.094)^2^	0.110 (0.087,0.132)^2^	0.100 (0.081,0.119)^2^	0.099 (0.080,0.119)^2^	0.120 (0.092,0.148)^2^
Model3	0.066 (0.040,0.093)^2^	0.116 (0.089,0.144)^2^	0.102 (0.081,0.124)^2^	0.100 (0.076,0.123)^2^	0.125 (0.095,0.156)^2^

Model 1 includes year only; Model 2 includes all the components of model 1 and education, PA, sedentary time, and income; Model 3 includes all the components of Model 2 and the urbanization index as a continuous variable.

^1^CI: Confidence interval.

^2^ *P* < 0.01.

### Associations between different levels of factors and different percentiles based on longitudinal QR analysis

Tables 3 and 4 show the estimated coefficients and the 95% CIs for the quantile models of male and female BMI, respectively. Higher PA and higher energy intake were associated with lower BMI in both genders. A higher educational level, higher income, and higher urbanization index were associated with a lower BMI in females. However, only the educational level and income had significant associations. Sedentary time had a positive association in the higher percentiles (50^th^, 75^th^, and 90^th^) but a negative association in the lower percentiles (10^th^ and 25^th^). A higher educational level, higher income, higher energy intake, and higher urbanization index were associated with higher BMI in males. For example, in the 95^th^ BMI percentile, one additional urbanization index unit resulted in an increase of 0.017 kg/m^2^ (95% CI: 0.004, 0.030).

**Table 3:** **Quantile regression Model 3: results obtained for the different percentiles of the male BMI (95% CI** ^**1**^ **)**

			**Coefficient**		
	**10th**	**25th**	**50th**	**75th**	**90th**
Intercept	18.655 (17.381,19.930)^2^	19.091 (18.162,20.020)^2^	18.977 (17.827,20.127)^2^	21.062 (19.952,22.172)^2^	22.374 (20.620,24.129)^2^
Age in 2011	0.010 (-0.008,0.028)	0.015 (0.001,0.030)^3^	0.041 (0.020,0.062)^2^	0.028 (0.012,0.045)^2^	0.009 (-0.016,0.035)
Education level					
None/primary/junior middle school	Reference	Reference	Reference	Reference	Reference
Senior school	0.231 (-0.434,0.897)	0.278 (-0.090,0.646)	-0.052 (-0.586,0.486)	0.184 (-0.205,0.573)	0.214 (-0.314,0.742)
College and above	-0.071 (-0.263,2.489)	0.490 (-1.820,2.800)	-0.093 (-1.390,1.122)	1.451 (-1.082,3.985)	0.789 (-1.993,3.570)
Income tertile					
Low income	Reference	Reference	Reference	Reference	Reference
Middle income	0.171 (-0.009,0.351)	0.042 (-0.125,0.210)	-0.003 (-0.157,0.150)	0.009 (-0.136,0.155)	0.188 (-0.077,0.454)
High income	0.187 (-0.035,0.409)	0.069 (-0.148,0.287)	0.207 (0.001,0.414)^3^	0.331 (0.140,0.523)^2^	0.319 (0.013,0.625)^3^
Energy intake (100 kcal/day)	0.006 (-0.005,0.017)	0.008 (0.000,0.016)^3^	0.009 (0.001,0.017)^3^	-0.002 (-0.012,0.008)	0.004 (-0.010,0.018)
Total physical activity (METs/d)	-0.002 (-0.005,0.001)	-0.006 (-0.008,-0.003)^2^	-0.003 (-0.007,0.000)^3^	-0.003 (-0.006,0.001)	-0.004 (-0.008,0.000)^3^
Sedentary time (hours/d)	-0.016 (-0.054,0.022)	0.002 (-0.030,0.034)	-0.011 (-0.039,0.018)	-0.013 (-0.051,0.025)	-0.031 (-0.084,0.021)
Urbanization index	0.017 (0.007,0.027)^2^	0.013 (0.004,0.022)^2^	0.013 (0.005,0.022)^2^	0.009 (0.001,0.017)^3^	0.017 (0.004,0.030)^2^

Note: Less than senior school education or greater is the reference group for education and the low income tertile is the reference group for income.

^1^CI: Confidence interval.

^2^ *P* < 0.01.

^3^ *P* < 0.05.

**Table 4:** **Quantile regression Model 3: results obtained for the different percentiles of the female BMI (95% CI** ^**1**^ **)**

			**Coefficient**		
	**10th**	**25th**	**50th**	**75th**	**90th**
Intercept	17.509 (16.280,18.738)^2^	17.194 (15.691,18.697)^2^	18.194 (17.111,19.278)^2^	19.607 (18.600,20.614)^2^	19.674 (18.566,20.783)^2^
Age in 2011	0.062 (0.042,0.082)^2^	0.076 (0.055,0.098)^2^	0.079 (0.063,0.095)^2^	0.073 (0.055,0.091)^2^	0.083 (0.062,0.103)^2^
Education level					
None/primary/junior middle school	Reference	Reference	Reference	Reference	Reference
Senior school	-0.529 (-1.108,0.049)	-0.273 (-0.943,0.397)	-0.735 (-1.228,-0.241)^2^	-0.951 (-1.596,-0.306)^2^	-0.250 (-1.202,0.701)
College and above	1.232 (-0.622,3.087)	0.201 (-1.572,1.974)	-0.706 (-2.444,1.031)	-1.555 (-3.894,0.784)	0.229 (-2.775,3.234)
Income tertile					
Low income	Reference	Reference	Reference	Reference	Reference
Middle income	-0.030 (-0.219,0.160)	0.009 (-0.138,0.157)	-0.022 (-0.137,0.092)	-0.041 (-0.172,0.089)	-0.007 (-0.278,0.264)
High income	-0.035 (-0.252,0.183)	-0.010 (-0.158,0.137)	0.008 (-0.175,0.192)	0.023 (-0.164,0.210)	-0.037 (-0.345,0.272)
Energy intake (100 kcal/day)	0.003 (-0.011,0.016)	0.003 (-0.009,0.014)	0.001 (-0.006,0.009)	0.004 (-0.004,0.012)	0.008 (-0.004,0.020)
Total physical activity (METs/d)	-0.002 (-0.006,0.001)	0.000 (-0.003,0.003)	-0.001 (-0.004,0.002)	-0.001 (-0.004,0.001)	-0.004 (-0.007,-0.001)^3^
Sedentary time (hours/d)	-0.005 (-0.047,0.037)	-0.002 (-0.041,0.036)	0.039 (0.007,0.071)^3^	0.019 (-0.021,0.059)	0.005 (-0.043,0.053)
Urbanization index	-0.003 (-0.015,0.008)	-0.006 (-0.019,0.008)	-0.003 (-0.012,0.006)	-0.004 (-0.012,0.004)	-0.004 (-0.013,0.005)

Note: Less than senior school education or greater is the reference group for education and the low income tertile is the reference group for income.

^1^CI: Confidence interval.

^2^ *P* < 0.01.

^3^ *P* < 0.05.

## Discussion

Using 20 years of data from the CHNS, we obtained evidence for an upward trend in the BMI distribution among Chinese adults aged 18–60 years. The LMS method was used to construct curves of the 10^th^, 25^th^, 50^th^, 75^th^, and 90^th^ percentiles, which indicated that BMI increased more in men than in women across the overall age distribution and in all percentiles. Moreover, there were larger increases in the upper percentiles than the lower percentiles among both genders, especially in women aged >40 years. However, male participants aged >30 years old had larger increases in their BMI than the younger age group, and the rate of increase slowed down for men aged >40 years. Thus, intervention strategies need to target high-risk groups, including women aged >40 years and men aged 30–40 years. Thus, the body composition change may be similar to that in other countries, but there are also unique features. Young-Ho Khang et al. reported that the BMI distribution exhibited shifts toward the right in both genders among Korean adults, but the shift was much more evident in men than women. Furthermore, the BMI tended to decrease among women aged 20–39 years [31]. Data obtained from Canadian surveys show that the BMI distribution has shifted to the right since the 1970s, especially among men, and men were more likely to be overweight than women [32].

Compared with previous analyses, the QR method we employed in the present study is more appropriate because the covariates are expected to shift more than the conditional mean of the BMI. We found that there were significant yearly increases in the BMI and these increases were greater in the upper percentiles compared with the lower percentiles. Time had different positive associations with men and women in different percentiles. In particular, men had larger coefficients than women in three percentiles (10^th^, 50^th^, and 75^th^). These results suggest that the increase in the prevalence of overweight/obesity in men was greater than that in women. Previous studies have documented increases in overweight and obesity with gender-related differences in the BMI trends [1,5,33]. Our findings provide new insights into gender-specific percentiles and we found that not all male of the BMI percentiles were higher than those of females. After adjusting for individual and urbanicity factors, the increase was significantly attenuated in the yearly changes for men, i.e., in the lower tail as well as the upper tail. This demonstrates that individual and community level factors influence the increase in male BMI. For women, after adjusting for individual factors, the yearly change in the increase in the BMI was also attenuated significantly. However, the yearly coefficients increased after adjusting for urbanicity factors. Thus, individual factors had the opposite association with the yearly change in the female BMI compared with the community level factors.

Other researchers have examined the effects of various factors on the trends in the BMI among Chinese subjects by applying traditional regression based on the mean, where most considered the individual level effects and few addressed the community level effects. In our QR-based study, we examined the associations among these covariates in different percentiles and we detected various changes in the trends in the BMI among men and women in China. This method provided more detailed information about how individual or community level factors might be associated with various BMI levels. Previous researchers have demonstrated that decreases in the PA levels play an important role in body weight increases [29,34]. Indeed, PA brings clear health and functional benefits that extend to all segments of the population [35,36]. Thus, many countries, such as the U.S.A., Canada, and China, have published guidelines to increase PA levels. However, although our analysis also showed that higher PA was associated with lower BMI in both genders, it had different effects on the BMI in various percentiles. In men, PA had the strongest negative association with BMI in the 25^th^ percentile, i.e., –0.006 kg/m^2^ (95% CI: –0.008, –0.003), and similar in other percentiles, i.e., about –0.003 kg/m^2^ (95% CI: –0.007, 0.000). By contrast, PA had the strongest negative association with female BMI in the 90^th^ percentile, i.e., -0.004 kg/m^2^ (95% CI: –0.007, –0.001). These results demonstrate that PA had a much stronger negative association with people who had a higher BMI. Thus, interventions might be more effective if greater encouragement could be focused on people in the upper BMI percentile. An inactive or sedentary lifestyle is known to be a distinct risk factor for numerous NCDs independent of PA [37,38]. In the present study, although sedentary time had a significant positive association with BMI in the upper percentile, there were negative associations in the lower percentiles. The detailed explanations of these associations need to be addressed in further studies. We found that energy intake had a positive association with BMI, but varied among different percentiles.

Finally, disparities are also related to educational attainment because Jones-Smith et al. found that lower educational attainment was associated with a higher BMI and an increased likelihood of being overweight among Chinese women, whereas higher educational attainment was a risk factor for being overweight among Chinese men [16]. In the present study, a higher educational level had a strong negative association with female BMI in the 75^th^ percentile. In addition, college level education and above had a positive association with BMI among women in the lower percentiles. This demonstrates that lower educational attainment was not associated with a higher BMI in all percentiles. Among men, higher educational attainment was negatively associated with BMI in some percentiles. It is possible that highly educated men have higher incomes and thus more social activities and opportunities for eating out compared with women. According to our analysis, higher income had a significant positive association with increased BMI in males. When they eat out, people tend to eat larger meals, consume more energy-dense foods, increase their alcohol intake, and reduce their vegetable and fruit consumption compared with eating at home [39,40].

Urbanicity and modernization have greatly affected the changing disease patterns seen in China [1]. Previous studies have detected various health disparities between urban and rural areas in China and other countries. However, among women living in communities with a lower initial urbanization score, a higher level of change in urbanicity was associated with a significantly higher likelihood of becoming overweight/obese during the follow-up period [3]. In the present study, we found that the urbanization index had different positive association with male BMI in various percentiles. Urbanization will continue unabated in China, thus it will continue to promote changes in dietary behavior, with increasing intake of edible oils, fried foods, and animal-derived foods, as well as declines in PA due to less occupational, domestic, and travel activity, and more sedentary time. This phenomenon is unlikely to reverse by itself. Lao et al. reported that obesity is leveling off overall in Guangdong province [41], but in China as a whole, it is very likely to continuing to increase due to economic disparities. Obesity-related diseases will continue to have major health implications in terms of shifts in the prevalence of NCDs and increased medical care costs [8,42]. In the present study, all of the percentile curves exhibited increasing trends from 1991 to 2011, and the levels increased more in the higher percentiles, which indicates that the BMI of people in the higher percentiles appeared to increase more rapidly. Thus, different intervention strategies should be adopted for men and women in different percentiles.

This study had several strengths. First, we incorporated individual and community level evidence into the models. Thus, an array of variables was considered in different percentiles, which facilitated a deeper and fuller analysis. Second, we used large-scale, longitudinal samples, which allowed us to examine the specific causes of the obesity epidemic in China. This study also had limitations. We did not consider the roles of sex hormones, which may respond to obesogenic environmental changes, thereby affecting the BMI. In addition, changes in the gut microbiome might help to explain the great increases in obesity during the past few years [6].

## Conclusions

Our analysis showed that there have been major increases in the BMI among adults in China over the past decades, where the increases in the BMI in the upper percentiles were greater than those in the lower percentiles in both genders, but especially in men. We used the QR analysis method to elucidate potential effects on the overall BMI distribution. A national strategy to tackle overweight/obesity is urgently needed to correct the shift in the BMI distribution in an efficient manner.

## Abbreviations

BMI: Body mass index
CHNS: China Health and Nutrition Survey
LMS: Lambda mu sigma
QR: Quantile regression
PA: Physical activity
NCDs: Non-communicable and chronic diseases
Q1: The 25th percentile
Q3: The 75th percentile

## References

[1] Gordon-Larsen P, Wang H, Popkin BM (2014). Overweight dynamics in Chinese children and adults. Obes Rev.

[2] Flegal KM, Carroll MD, Kit BK, Ogden CL (2012). Prevalence of obesity and trends in the distribution of body mass index among US adults, 1999-2010. JAMA.

[3] Jones-Smith JC, Popkin BM (2010). Understanding community context and adult health changes in China: development of an urbanicity scale. Soc Sci Med.

[4] Stevens GA, Singh GM, Lu Y, Danaei G, Lin JK, Finucane MM (2012). National, regional, and global trends in adult overweight and obesity prevalences. Popul Health Metr.

[5] Li XY, Jiang Y, Hu N, Li YC, Zhang M, Huang ZJ (2012). Prevalence and characteristic of overweight and obesity among adults in China, 2010. Chinese J Prev Med.

[6] Ng M, Fleming T, Robinson M, Thomson B, Graetz N, Margono C (2014). Global, regional, and national prevalence of overweight and obesity in children and adults during 1980-2013: a systematic analysis for the global burden of disease study 2013. Lancet.

[7] World Health Organization (2009). Global health risks: mortality and burden of disease attributable to selected major risks.

[8] Zhang J, Shi X, Liang X (2013). Economic costs of both overweight and obesity among Chinese urban and rural residents in 2010. Chinese J Epidemiol.

[9] Bottai M, Frongillo EA, Sui X, O’Neill JR, McKeown RE, Burns TL (2014). Use of quantile regression to investigate the longitudinal association between physical activity and body mass index. Obesity (Silver Spring).

[10] Popkin BM (2014). Synthesis and implications: China’s nutrition transition in the context of changes across other low- and middle-income countries. Obes Rev.

[11] Swinburn BA, Sacks G, Hall KD, McPherson K, Finegood DT, Moodie ML (2011). The global obesity pandemic: shaped by global drivers and local environments. Lancet.

[12] Popkin BM (2008). Will China’s nutrition transition overwhelm its health care system and slow economic growth?. Health Aff (Millwood).

[13] Reynolds K, Gu D, Whelton PK, Wu X, Duan X, Mo J (2007). Prevalence and risk factors of overweight and obesity in China. Obesity (Silver Spring).

[14] Popkin BM, Gordon-Larsen P (2004). The nutrition transition: worldwide obesity dynamics and their determinants. Int J Obes Relat Metab Disord.

[15] Ng SW, Howard AG, Wang HJ, Su C, Zhang B (2014). The physical activity transition among adults in China: 1991-2011. Obes Rev.

[16] Shankar B (2010). Obesity in China: the differential impacts of covariates along the BMI distribution. Obesity (Silver Spring).

[17] Jones-Smith JC, Gordon-Larsen P, Siddiqi A, Popkin BM (2012). Emerging disparities in overweight by educational attainment in Chinese adults (1989-2006). Int J Obes (Lond).

[18] Subramanian SV, Perkins JM, Ozaltin E, Davey Smith G (2011). Weight of nations: a socioeconomic analysis of women in low- to middle-income countries. Am J Clin Nutr.

[19] Dahly DL, Gordon-Larsen P, Popkin BM, Kaufman JS, Adair LS (2010). Associations between multiple indicators of socioeconomic status and obesity in young adult Filipinos vary by gender, urbanicity, and indicator used. J Nutr.

[20] Razak F, Corsi DJ, Subramanian SV (2013). Change in the body mass index distribution for women: analysis of surveys from 37 low- and middle-income countries. PLoS Med.

[21] Neuman M, Kawachi I, Gortmaker S, Subramanian SV (2013). Urban-rural differences in BMI in low- and middle-income countries: the role of socioeconomic status. Am J Clin Nutr.

[22] Geraci M, Bottai M (2007). Quantile regression for longitudinal data using the asymmetric Laplace distribution. Biostatistics.

[23] Zhang B, Zhai FY, Du SF, Popkin BM (2014). The China health and nutrition survey, 1989-2011. Obes Rev.

[24] Du S, Mroz TA, Zhai F, Popkin BM (2004). Rapid income growth adversely affects diet quality in China–particularly for the poor!. Soc Sci Med.

[25] Zhai F, Guo X, Popkin BM, Ma L, Wang Q, Yu W (1996). Evaluation of the 24-hour individual recall method in China. Food Nutri Bull United Nations Uni.

[26] Zhai FY, Du SF, Wang ZH, Zhang JG, Du WW, Popkin BM (2014). Dynamics of the Chinese diet and the role of urbanicity, 1991-2011. Obes Rev.

[27] Ng SW, Norton EC, Popkin BM (2009). Why have physical activity levels declined among Chinese adults? Findings from the,1991–2006 China health and nutrition surveys. Soc Sci Med.

[28] Monda KL, Adair LS, Zhai F, Popkin BM (2008). Longitudinal relationships between occupational and domestic physical activity patterns and body weight in China. Eur J Clin Nutr.

[29] Monda KL, Gordon-Larsen P, Stevens J, Popkin BM (2007). China’s transition: the effect of rapid urbanization on adult occupational physical activity. Soc Sci Med.

[30] Adair LS, Gordon-Larsen P, Du SF, Zhang B, Popkin BM (2014). The emergence of cardiometabolic disease risk in Chinese children and adults: consequences of changes in diet, physical activity and obesity. Obes Rev.

[31] Khang YH, Yun SC (1998). Trends in general and abdominal obesity among Korean adults: findings from, 2001, 2005, and 2007 Korea national health and nutrition examination surveys. J Korean Med Sci.

[32] Gotay CC, Katzmarzyk PT, Janssen I, Dawson MY, Aminoltejari K, Bartley NL (2013). Updating the Canadian obesity maps: an epidemic in progress. Can J Public Health.

[33] Xi B, Liang Y, He T, Reilly KH, Hu Y, Wang Q (2012). Secular trends in the prevalence of general and abdominal obesity among Chinese adults, 1993-2009. Obes Rev.

[34] Ng SW, Popkin BM (2012). Time use and physical activity: a shift away from movement across the globe. Obes Rev.

[35] Popkin BM, Du S, Zhai F, Zhang B (2010). Cohort profile: the China health and nutrition survey–monitoring and understanding socio-economic and health change in China, 1989–2011. Int J Epidemiol.

[36] Zhang J, Chaaban J (2013). The economic cost of physical inactivity in China. Prev Med.

[37] Thorp AA, Owen N, Neuhaus M, Dunstan DW (2011). Sedentary behaviors and subsequent health outcomes in adults a systematic review of longitudinal studies, 1996-2011. Am J Prev Med.

[38] Owen N, Healy GN, Matthews CE, Dunstan DW (2010). Too much sitting: the population health science of sedentary behavior. Exerc Sport Sci Rev.

[39] O’Dwyer NA, Gibney MJ, Burke SJ, McCarthy SN (2005). The influence of eating location on nutrient intakes in Irish adults: implications for developing food-based dietary guidelines. Public Health Nutr.

[40] Vandevijvere S, Lachat C, Kolsteren P, Van Oyen H (2009). Eating out of home in Belgium: current situation and policy implications. Br J Nutr.

[41] Lao XQ, Ma WJ, Sobko T, Zhang YH, Xu YJ, Xu XJ (2015). Overall obesity is leveling-off while abdominal obesity continues to rise in a Chinese population experiencing rapid economic development: analysis of serial cross-sectional health survey data 2002-2010. Int J Obes (Lond).

[42] Zhao W, Zhai Y, Hu J, Wang J, Yang Z, Kong L (2008). Economic burden of obesity-related chronic diseases in Mainland China. Obes Rev.

